# The Role of Mindfulness and Emotional Intelligence on Adolescents’ Well-Being and Secondary School Achievement

**DOI:** 10.1177/00332941241301638

**Published:** 2024-11-18

**Authors:** Inês Vieira, Luísa Faria

**Affiliations:** Faculty of Psychology and Education Sciences, 26706 University of Porto, Center for Psychology at University of Porto, Rua Alfredo Allen, Porto, Portugal

**Keywords:** Mindfulness, trait-emotional intelligence, well-being, school achievement, adolescence

## Abstract

The present study aimed to determine how mindfulness relates to school achievement and well-being in adolescence and whether this relationship might be mediated by emotional intelligence (EI). Apart from examining the relationship between constructs, moderation interaction effects of gender were also tested. A sample of 597 Portuguese adolescents aged 15–17 years old (*M*_age_ = 16.9; *SD* = 1.11; 49.9% males), enrolled in secondary education, completed self-report measures of mindfulness (CAMM), emotional intelligence (TEIQue-ASF), and well-being (SWLS) in their Portuguese versions. Findings confirmed a positive and statistically significant relationship between mindfulness and EI. Regression analyses indicated that mindfulness reduced its influence on well-being and on school results when EI was added to the model, confirming the mediation hypothesis. Results indicated a positive and statistically significant relationship between EI and well-being. Adolescents with higher traits of mindfulness did not report better school results neither greater well-being. Variations across gender have not been observed. This study discloses several limitations which require a careful examination of the results. The findings are discussed considering the potential advantages of including social and emotional competencies in educational settings as a complement to the school curriculum.

## Introduction

Schoolwork pressure resulting in states of anxiety, depressive symptoms, or lower levels of life satisfaction in youth, comes from peers, parents, teachers, and personal expectations to achieve good grades, performing well in exams and being admitted to post-secondary education (OECD, 2019). In Portugal, research reported that 42.2% of adolescents reveal moderate to severe symptoms of depression, and reflecting the European data, females report worse mental health indicators than males (European Commission, 2018; Pereira et al., 2022). The work to buffer negative emotional outcomes in the facing of adverse situations has come along with personal protective factors of social and emotional nature that act as supportive elements when dealing with emotional challenges (OECD, 2019), such as mindfulness and emotional intelligence (EI), which have gathered scientific interest over the past decades.

### Mindfulness

Mindfulness is described as “the state of being attentive to and aware of what is taking place in the present” (Brown & Ryan, 2003, p. 822). Apart from a skill that can be learnt through regular practice, the literature also acknowledges mindfulness as a trait, that is, “an inherent, natural capacity of the human organism” (Brown & Ryan, 2004, p. 246). Escalating schoolwork stress in early stages as high school has potentiated mindfulness research in school settings (Broderick, 2021), corroborating its benefits on mental health, stress and anxiety reduction, and socioemotional impact on children and adolescents (Caballero et al., 2019; Monsillion et al., 2023; Pinto & Carvalho, 2019). However, the *Westernization* of mindfulness in various contexts has brought to discussion its actual meaning and effectiveness of its applicability, as originally in Buddhist culture the concept is much broader defined (Kabat-Zinn, 2016). Recent studies in the Portuguese context concluded that high levels of dispositional mindfulness can positively impact school achievements, thus fostering academic success, while students with lower levels may be at disadvantage (Limpo et al., 2022). Monsillion and colleagues (2023) conducted a systematic review suggesting that mindfulness may benefit emotional and behavioral regulation impacting school environment and well-being, despite identifying discrepancies in terms of methodology. Overall, there seems to be a consensus on a positive relationship between mindfulness and academic success both on correlational and experimental research, although limitations for the latest recognize the need of investing on more robust studies on mindfulness as a trait-like construct (e.g., Birtwell et al., 2019; Magalhães et al., 2022). Parallelly with positive academic outcomes, a growing body of literature acknowledges the effects of mindfulness on well-being. As adolescence is a critical period of human development affected by personal, social, and physiological transformations, the incidence of emotional problems impacting psychological well-being increases (Olson et al., 2021). Findings have shown that dispositional mindfulness correlates negatively with several psychological pathologies and mental health issues, such as depression, anxiety, and difficulties in emotion regulation (Kabat-Zinn, 2003), and correlates positively with self-esteem and life satisfaction, which are fundamental factors of emotional balance and well-being (Brown & Ryan, 2003; Kabat-Zinn, 2016). Brain research has evidenced that mindfulness can influence development in youth by empowering self-awareness, self-regulation, resilience, and empathy, as crucial elements to better handle stressful situations, emotions, aggression, impulsivity, and executive functions (Snel, 2019). This connection between mindfulness and well-being seems to be greater for females (Bluth et al., 2017; Carsley et al., 2018; Moreira et al., 2018), although some studies have not found variations across gender (Johnson et al., 2017; Magalhães et al., 2022).

### Emotional Intelligence

In similarity with mindfulness, and playing a role on well-being, EI research in educational settings has been particularly remarked for the impact of social and emotional competency in school success (Costa & Faria, 2015). EI has been defined as “the ability to monitor one’s own and others’ feelings and emotions, to discriminate among them, and to use this information to guide one’s thinking and actions” (Salovey & Mayer, 1990, p. 189), and has also been associated to personality traits and dispositional factors which can be evaluated through self-report measures (Petrides & Furnham, 2001; Zadorozhny et al., 2024). Apart from conceptualization variances, a wide range of literature found EI to be a predictor of several outcomes and relate positively with multiple variables. Within educational context, EI has been noted as a predictor of academic achievement (Quílez-Robres et al., 2023), and as a strategy towards school success (Costa & Faria, 2015; Moutinho et al., 2019; Pérez-González et al., 2020), in addition to other relevant outcomes as school adaptation, academic performance, and academic engagement (Costa & Faria, 2020; Martín et al., 2021). Studies suggest that more conscious adolescents can manage emotions effectively and better adapt to schoolwork, thus coping with expectations, anxiety, and school demands (Martín et al., 2021). Moreover, higher traits of emotional self-efficacy in adolescents were found to correlate negatively with school burnout (Fiorilli et al., 2020), supporting the relationship between EI and well-being already endorsed in the literature. Positive correlations with well-being have been found within the Portuguese context (Costa et al., 2021; Moutinho et al., 2019), in similarity with international studies (e.g., Extremera & Fernández-Berrocal, 2006; Foster et al., 2018; Ioannidou & Argyriadi, 2024; Molero Jurado et al., 2021; Walter et al., 2021). EI has been related to emotion regulation and adaptive behavior (Goleman, 1995) which can evolve to states of emotional and intellectual growth through a greater emotional knowledge and understanding (Mayer & Salovey, 1997). In the systematic review of Llamas-Días and colleagues (2022), the authors observe a positive and significant relationship between EI and subjective well-being in adolescence for all studies including self-report measures, both for ability and mixed models. Findings suggest that the implementation of EI training in educational context can prevent emotional disorders and contribute to mental health in adolescence, while according to Piqueras and colleagues (2020), trait EI along with mindfulness skills can act as protective factors on the development of psychopathologies. Yet, despite young girls reporting worse mental health indicators, the evidence is not consistent on the role of EI across gender (Cabello et al., 2016; Costa & Faria, 2020).

### Mindfulness and Emotional Intelligence

Regarding mindfulness, EI and well-being, prior studies have considered the relation between the three constructs. Chu (2010) not only concluded that more mindful individuals revealed greater EI, for being better at regulating attention and energy, but they also reported to feel less stress and negative mental health when compared to the control group. Similar observations were emphasized by Wang and Kong (2014) who examined the mediator role of EI between mindfulness and two indicators of subjective well-being. The results demonstrated that EI partially mediated this relation, and while gender differences did not impact results, it was noted that individuals with higher levels of mindfulness who were not students experienced greater life satisfaction than individuals with high levels of mindfulness who were simultaneously studying (Wang & Kong, 2014). The connection between mindfulness and EI in the pre-existing literature claims for further research and has taken researchers to explore how both variables relate to each other (Rodríguez-Ledo et al., 2018). The mechanisms underlying mindfulness, such as the inner attention to psychological states, resemble the construct of EI, and studies indicate that mindfulness strengthens the development of key abilities and competencies of EI (Miao et al., 2018). In educational context, both have been positively related to attentional aspects, reduced ADHD indicators, empathy to social and interpersonal problems, decreased aggressiveness and impulsivity, despite worse attentional factors have been found in adolescents when compared to children or adults (Rodríguez-Ledo et al., 2018). In fact, socioemotional programs or interventions implemented in school settings have revealed promising results, supporting its mental health benefits, stress and anxiety reduction, and positive impact on children and adolescents (Pinto & Carvalho, 2019). The successful implementation allied with scientific evidence has potentiated a growth of such interventions, many combining mindfulness training with socio-emotional learning based on CASEL’s framework, although researchers identify limitations, considering that further investigation is needed to understand the mechanisms underlying the effects of such practice (Limpo et al., 2023). Compelling this need there seems to be an increase on the foundation of mindfulness as a trait while regular practice is administrated (Limpo et al., 2023; Quaglia et al., 2016). In adolescence, both EI and mindfulness have been independently related to well-being and mental health as their interactive effect remains reasonably unknown (Teal et al., 2018), although previous studies seem to indicate a correlation (Testa & Sangganjanavanich, 2016). However, recent literature suggests a complementary approach of trait EI and dispositional mindfulness, rather than assessing both separately, with greater intervention effects for adolescents (Mestre et al., 2019). Yet, some studies also recognize the need of caution when looking through practical interventions, alerting that the effects have reported inconsistency and failure to mirror clear conclusions (Pickerell et al., 2023). In sum, there seems to be an agreement on the importance of additional research advances.

### The current study

Based on these open questions reported in the literature, the main purpose of this study was to explore the concepts of dispositional mindfulness, trait EI and well-being as well as examining how the three constructs are interconnected in our target youth. We intended to perceive the relevance of mindfulness and individual emotional competencies to school achievement and well-being. Secondly, we explored the effects of mindfulness on well-being and school results under mediation of EI. Thirdly, we observed whether mindfulness and well-being variated across students’ gender. Accordingly, the main study’s hypotheses are: h1) Adolescents with stronger traits of mindfulness report better school results and greater well-being, although this effect is mediated by emotional intelligence; h2) The relationship between mindfulness and well-being is moderated by gender. While our study takes part in a complex task of bringing clarity to the phenomenon underlying the relationship between these variables, we acknowledge its brief contribution to the field through the examination of a niche reality that may not be captured equally for a broader population. Yet, we highlight the exploratory nature of this research as an intention of contributing to the current debate on moving towards a more socio-emotional oriented education.

## Method

### Participants

The sample was composed of 597 Portuguese adolescents enrolled in secondary education (*M*_age_ = 16.9; *SD* = 1.11). Male students accounted for 49.9% of the sample. 31.2% of students were 10^th^ graders, 32.8% were 11^th^ graders and 36.0% were 12^th^ graders. Non-probability sampling was used to obtain the research participants. The absence of legal guardian permission was used as exclusion criteria.

### Procedure

Data collection implied the fulfillment of a survey available in digital format using students’ personal devices (smartphones, tables or laptops). To ensure confidentiality and independent response the participants completed the survey during normal class schedule under supervision of the teacher and one researcher. Devices have not been shared at any moment. The respondents were provided with written instructions describing the procedure and were informed of their voluntary and anonymous participation with the possibility of withdrawing from the study at any point in time with no penalties. There was no space to introduce personal identification and all records were kept confidential, exclusively for research purposes. The project protocol was approved by the Ethics Committee of University of Porto. The empirical work was preceded by schools’ authorization, required teachers’ availability, and written informed consent of legal guardians (for students under 18). No benefits or incentives were offered. The use of CAMM, TEIQue-ASF and SWLS was proceeded by authorization of their respective authors. Besides an introductory framework explaining the purpose of the study, a section of the survey was centered on sociodemographic and academic features. The remaining part was dedicated to three instruments measuring mindfulness, emotional intelligence and well-being. The fulfillment of the survey took 7–10 minutes, and the consultation period was 6 months. Academic achievement was based on students’ self-reported overall grade point average (GPA; grade range 0–20), which we found to be the most appropriate collection method for our study. To ensure the best screening and quality of the data, each survey was reviewed independently to eliminate potential response bias and improve the credibility of our findings. This work allowed us to identify and remove responses from individuals who either did not match our target audience criteria (for instance, aged over 93) or did not answer the questions thoughtfully (for instance, participants who straight-lined or whose survey responses projected a geometric pattern). After conclusion of this work, a total of 597 responses were considered to proceed with the data analyses. Although acknowledging that not all bias is preventable, we ensured the best practices to prevent them from negatively affecting the results of our research.

### Measures

#### Child and Adolescent Mindfulness Measure (CAMM)

For the assessment of trait mindfulness participants completed the CAMM (Greco et al., 2011) in its Portuguese version (Cunha et al., 2013), a single-factor self-report scale of 10 items negatively worded and reversed-scored (e.g., “I push away thoughts that I don’t like”), so that higher scores represent greater mindfulness (Greco et al., 2011). The participants rate how often each item is true using a 5-point Likert scale, varying from zero (never true) to 4 (always true). This measure was suitable to the age range of our study (15–18 years old; Cunha et al., 2013) and has scored positive correlations with academic competence, social skills, and quality of life (Goodman et al., 2017).

A Confirmatory Factor Analysis (CFA) was performed for the CAMM, χ^2^/df = 5.259; CFI = .918; GFI = .943; RMSEA = .083, indicating an acceptable model fit. The internal consistency was reported .81 in line with previous findings, for example, the English (α = .81; Greco et al., 2011), or the Dutch versions (α = .80; Goodman et al., 2017). The Portuguese version also presented adequate internal consistency (α = .80) and test-retest reliability (α = .46) for adolescents aged 15–18 years old.

#### Trait Emotional Intelligence Questionnaire – Adolescent Short Form (TEIQue-ASF)

The participants completed the TEIQue-ASF (Carvalho & Lopes, 2013; Petrides et al., 2016; Portuguese version of Carvalho & Lopes, 2013), for measurement of EI. This measure was developed for adolescents aged 13–17 years old with 30 statements that measure the global trait of EI (e.g., “I pay a lot of attention to my feelings”) in a 7-point Likert scale, ranging from 1 (disagree completely) to 7 (agree completely), and compose four different factors: emotionality, self-control, sociability, and well-being (Petrides, 2009).

CFA for the TEIQue-ASF, χ^2^/df = 3.553; CFI = .767; GFI = .801; RMSEA = .081; SRMR = .088 (SRMR ≥.08; Hair et al., 2022), indicated a poor model fit. The construct reported good scores of internal consistency and discriminant validity, although indicating low convergent validity with values of Average Variance Extracted (AVE) inferior to .50 (Table 1). The adjustment of the model implied the correlation of errors 2 and 3, 2 and 8, 10 and 13, 24 and 26, and 22 and 25. The reliability score ranged between .72 (significant) for the *emotionality* dimension and .82 (very good) for the *well-being* dimension (Hair et al., 1998). Petrides and colleagues (2016) emphasize that the internal consistency of the global score of the scale usually exceeds .80, which was confirmed by Piqueras et al. (2020) for the Spanish version (.85), although other authors noted psychometric inconsistencies linked to the adolescent version of this measure (Mavroveli & Siu, 2012), and due to cultural nuances (Feher et al., 2019; Walter et al., 2021). Zadorozhny and colleagues (2024), for example, reported poor reliability on the *self-control* dimension in their study.

**Table 1.** Convergent and Discriminant Validity of TEIQue-ASF.

	Cronbach’s alpha	CR	AVE	MSV	MaxR(H)	Emo	Self-C	Soc	WB
Emo	.72	.71	.25	.24	.74	.50			
Self-C	.74	.74	.32	.21	.76	.36***	.57		
Soc	.72	.72	.31	.21	.73	.39***	.31***	.55	
WB	.82	.82	.44	.24	.86	.49***	.46***	.46***	.67

*p* ≤ .001 *CR* construct reliability; *AVE* average variance extracted; *MSV* maximum shared variance; *MaxR(H)* maximum reliability; *Emo* emotionality; *Self-C* self-control; *Soc* sociability; *WB* well-being.

#### Satisfaction With Life Scale (SWLS)

Participant’s well-being was assessed through the SWLS (Diener et al., 1985), validated to the Portuguese context by Neto (1993). The SWLS is a self-reported instrument to evaluate subjective well-being consisting of 5 statements, answered in a 7-point Likert scale (from strongly disagree - 1 to strongly agree - 7), which appraise an individual’s sense of satisfaction with life (e.g., “I am satisfied with my life”; Diener et al., 1985). The instrument was found to highly correlate with other measures of subjective well-being (Diener et al., 1985).

CFA for the SWLS χ^2^/df = 1.466; CFI = .998; GFI = .995; RMSEA = .028 indicated a good model fit. The reliability score was reported .84, being consistent with the original English version which reported alpha coefficients ranging from .79 to .89 (Diener et al., 1985). In Portugal, within the adolescent population, Neto (1993) confirmed a good internal consistency coefficient of .78, sustaining the international evidence.

### Data Analysis

The 29.0 version of IBM SPSS and AMOS supported the statistical analyses which included descriptive and inferential statistics for theory testing. For the latest, Pearson’s *r* was computed between correlated qualitative variables, one sample *t* test for mean comparisons, one-way analysis of variance (ANOVA), and multivariate analysis of variance (MANOVA), path analysis to examine the multiple causal pathways between variables, and moderation and mediation interaction effects. The significance level was fixed at α ≤ .05. A path analysis and model testing followed as second step. We considered as fit indices Chi-square (*x*^2^), Confirmatory Factor Index (CFI), Goodness of Fit (GFI) and Root Mean Square Error of Approximation (RMSEA), based on the reference values *x*2/df > .05, CFI ≥.90, GFI ≥.90 and RMSEA <.08 suggested by Hooper et al. (2008) and Kline (2023). CFA was selected in our study to validate all measures as their factor structure and internal reliability have been robustly recognized in the literature, as well as their good psychometric properties, particularly within adolescent populations (e.g., Cunha et al., 2013; Fiorilli et al., 2020; Usán Supervía et al., 2022).

## Results

### Preliminary Analyses

A primary step of descriptive analyses disclosed |sk| ≤ 2 and |ku| ≤ 2, thus within the reference values (Kline, 2023). Cronbach’s Alphas were calculated as reliability indices of the measures (Table 2).

**Table 2.** Descriptive Statistics and Reliability Analysis of CAMM, TEIQue-ASF and SWLS (*N* = 597).

Measure	Min	Max	Mean	SD	sk	ku	Cronbach’s alpha
CAMM	1.00	7.00	3.99	1.11	.08	−.26	.81
TEIQue-ASF	2.16	6.83	4.41	.72	.06	.03	.88
SWLS	1.20	5.00	3.38	.78	−.11	−.38	.84

*sk* skewness; *ku* kurtosis.

### Path Analysis

As per the study objectives, path analysis was used to understand how mindfulness relates to school results and well-being of adolescent students and whether this relationship might be mediated by EI and moderated by gender differences.

The values of satisfaction with life (3.38) were significantly higher than the middle value of the scale, t (597) = 12.072, *p* < .001. For mindfulness (3.99), the score equaled the middle value of the scale, t (597) = −.081, *p* = .935. Except for the *self-control* dimension, t (597) = .886, *p* = .376, all remaining values of EI were significantly higher than the middle point of the scale, *p* < .00. Participants reported greater levels of *sociability* and lower levels of *self-control*. Apart from the differences between *sociability* and *well-being* (*p* = 1.000), all remaining were significant.

The correlation coefficients between EI dimensions were significant, positive, and moderate as expected within the dimensions of one same construct. The correlations between EI, mindfulness and satisfaction with life were also significant and positive, although weak (Table 3).

**Table 3.** Correlation Matrix Between TEIQue-ASF Dimensions, SWLS and CAMM.

	Emotionality	Self-control	Sociability	Well-being	SWLS
Emotionality	---				
Self-control	.29**				
Sociability	.30**	.21**			
Well-being	.47**	.38**	.33**		
SWLS	.36**	.27**	.26**	.73**	
CAMM	.37**	.31**	.19**	.36**	.23**

*p < .05 **p < .01 ***p < .001.

**Figure 1.**
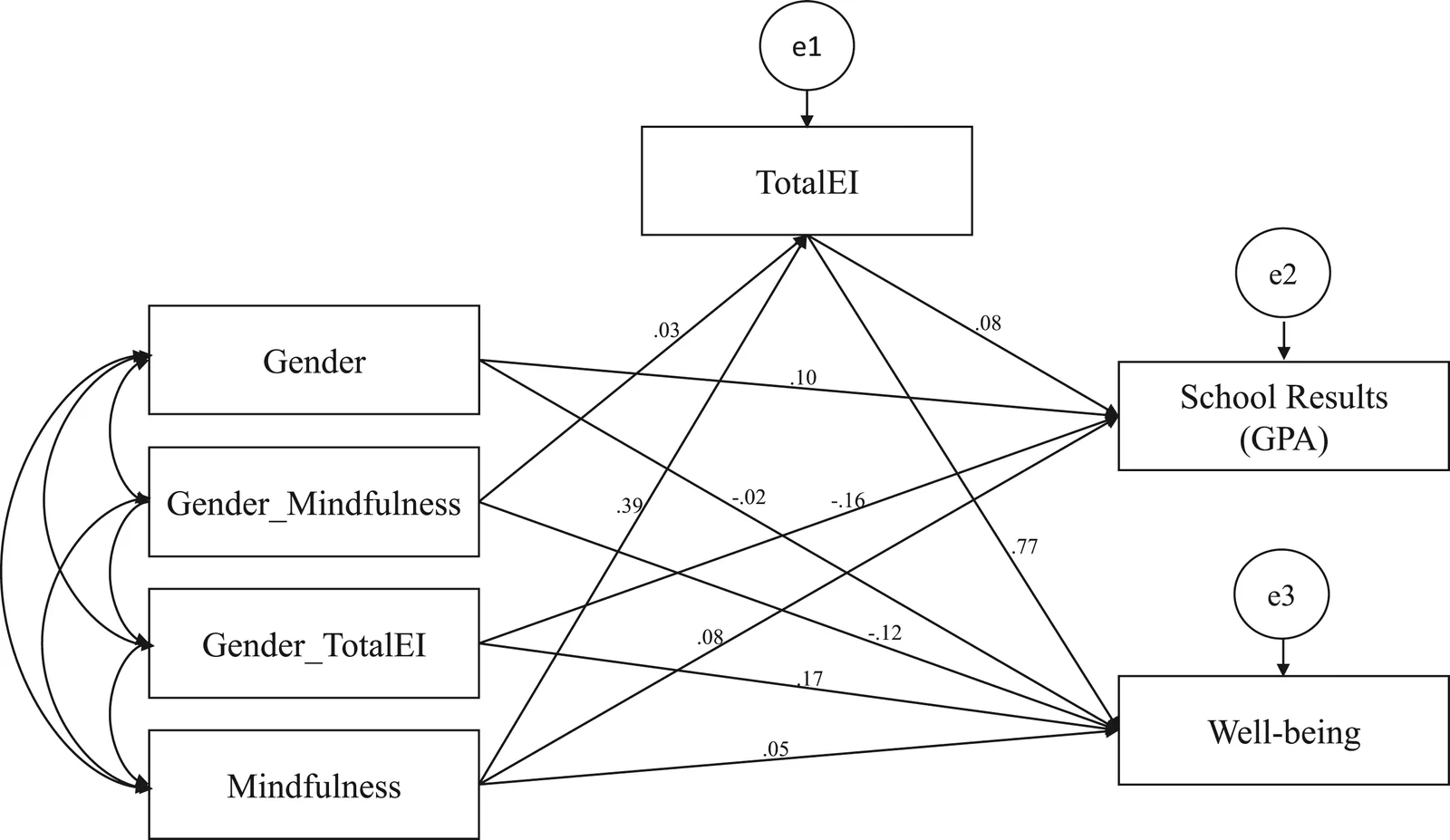
Standardized path estimates of the relationship between variables.

The path analysis resulted on an output path diagram confronting the theorized model with the existing effects (Figure 1). Standardized and unstandardized statistics may be consulted in Table 4.

**Table 4.** Standardized and Unstandardized Statistics.

			Unstandardized Coefficient	β	Standard Error	Critical ratio	p
totalEI	<---	mindfulness	.25	.39	.04	7.05	***
totalEI	<---	gender_mindfulness	.01	.03	.01	.68	.50
school results	<---	mindfulness	.16	.08	.10	1.58	.11
school results	<---	totalEI	.27	.08	.15	1.84	.07
well-being	<---	totalEI	1.27	.77	.05	26.42	***
well-being	<---	mindfulness	.05	.05	.04	1.22	.22
well-being	<---	gender_totalEI	.09	.17	.08	1.04	.30
well-being	<---	gender_mindfulness	−.07	−.12	.06	−1.10	.27
school results	<---	gender	.45	.10	1.04	.43	.67
well-being	<---	gender	−.04	−.02	.36	−.12	.91
school results	<---	gender_totalEI	−.16	−.16	.23	−.69	.49

***p ≤ .001.

For mediation analyses (Table 5), the model explained 3.4% of the total variance of school results and the total indirect effects were .114, thus statistically significant, IC 95% (.014; .092, *p* = .007). Mindfulness reduced its influence on school results (r = .14) when EI was added to the model (r = .09). Additionally, the model explained 64.5% of the total variance of well-being and the total indirect effects were .375, thus statistically significant, IC 95% (.280; .401, *p* < .001). Mindfulness reduced its influence on well-being (r = .36) when EI was added to the model (r = .02). A positive and statistically significant relationship between mindfulness and EI was observed (B = .252, *p* < .001). EI was positively associated with school results and well-being, although not statistically significant for the first (B = .271, *p* = .065), but statistically significant for well-being (B = 1.269, *p* < .001). The relationship between mindfulness and school results (B = .158, *p* = .114) and well-being (B = .051, *p* = .223), were both non-significant. For the latest, moderation analysis of gender was also reported non-significant (B = −.067, *p* = .273).

**Table 5.** Regression Analyses for School Results and Well-Being.

	School Results					
	Model 1		Model 2		Model 3	
	EI		School Results		School Results	
Independent variables	B	SE	B	SE	B	SE
Mindfulness	.27***	.02	.32***	.02	.21*	.10
Emotional intelligence	---	---			.42**	15
R2					.03***	
F					10.13	
CI 95%					.01; .09	
	Well-being					
	Model 1		Model 2		Model 3	
	EI		Well-being		Well-being	
Independent variables	B	SE	B	SE	B	SE
Mindfulness	.28***	.02	.40***	.04	.02	.03
Emotional intelligence	---	---			1.34***	.05
*R* ^2^					.65***	
F					541.54	
CI 95%					.28; .40	

*p ≤ .05 **p ≤ .01 ***p ≤ .001.

## Discussion

In a demanding phase of the lifespan such as adolescence, students face a growing concern about the future and an increase of academic responsibilities that escalate complexity every year. Finding a balance between mastering academic skills and emotional well-being can become challenging, and socioemotional competencies in educational settings must be adequately met as they can contribute to school success and mental health, supporting students expressing themselves and crossing positively through their psychological states. In the present study, we examined the relationship between dispositional mindfulness, trait EI and well-being. Moreover, we explored the interaction effects of mindfulness on well-being and school results, under mediation of EI, and gender variations on mindfulness and well-being.

Adolescents with stronger traits of mindfulness did not report better school results neither greater well-being, diverging from previous research acknowledging a positive relationship between mindfulness and both school results (Caballero et al., 2019; Limpo et al., 2022; Monsillion et al., 2023) and well-being (Brown & Ryan, 2003; Kabat-Zinn, 2016; Pinto & Carvalho, 2019). Such divergency might be grounded on four potential reasons that compel a careful evaluation of our results. First, as we examined trait self-report mindfulness, it is prudent to note that our sample did not participate of any previous meditation experience, and positive associations have been generally related to practical interventions in school settings (e.g., Magalhães et al., 2022). Future studies assessing trait mindfulness should consider the use of behavioral data to understand underlying elements which have not been studied in this paper. Second, the brain’s default mode network of mindfulness practitioners has been associated to less ruminative thoughts, less mind-wondering, and less distraction, although individuals scoring high state mindfulness may not necessarily disclose high trait mindfulness (Treves et al., 2024), which might be linked to distinct interpretations of questionnaires. Third, the theoretical implications of implementing an original Buddhist concept in a Western educational context has been discussed as a tension of cultural values in the current literature and may also explain these effects. The Eastern view of mindfulness as an evolutive practice of cognitive processes and emotional consciousness, not only contrasts with the Western psychological tool to face negative emotional experiences, but also with the challenges of adolescents applying a concept rooting a different cultural setting into everyday school life. These cultural variations may lead to different interpretations of the concept which deviate from the main goal of their applicability and influence their effectiveness, for instance, as emotions and traits manifest differently across cultures, it is expected to observe disparities between Western adolescents (from individualistic societies focused on the self) and Eastern adolescents (from collectivistic societies protective of social harmony). Four, our sample demographic features, their age range and school pressure, must be accounted for as trait mindfulness may be refined over time in late adolescence and adulthood, but also with the end of secondary education as a period of psychological overload, noting that previous research related higher trait mindfulness to individuals who were not studying (Wang & Kong, 2014). The replication of this study with an older sample is advised and could carry different outcomes. Factually, mindfulness cultural nuances and its dual-faceted nature require further investigation to align its conceptualization and bring empirical consensus. The results demonstrated that as mindfulness increases the levels of EI tend to be higher, adding consensus to existing evidence indicating a positive relationship between both (Mestre et al., 2019; Miao et al., 2018; Testa & Sangganjanavanich, 2016), potentially explained by their intersection on socioemotional baseline mechanisms. Although we could not verify a relationship between EI and school results, as expected per the existing literature relating EI to greater academic outcomes (e.g., Quílez-Robres et al., 2023), we verified that adolescents with higher levels of trait EI reported greater well-being, possibly suggesting that these students are able to better adopt behavioral strategies to cope with stressful situations and minimize negative mental health consequences. Furthermore, we observed the correlation between mindfulness and school results to be greater when EI was added to the model. Similar conclusions were verified for well-being, corroborating the mediation effects (h1). Noting, however, that fluctuations on total variance were found between well-being and school results. Recent findings suggest that students with higher consciousness of their evolutive emotional competencies are keener on positively face new experiences, but not necessarily reveal better school results (Costa & Faria, 2023), which may to some extent explain these differences. Additionally, the mediation results may indicate a higher predictive effect of EI over mindfulness, which partially reduces the influence of the latest, and can also be explained according to previous literature linking mindfulness to attention and acceptance of emotional experiences while EI involves flexibility to understand and regulate emotional states, and students may use this knowledge to persevere in daily moments of mental distress, controlling feelings of impulse and frustration. Yet, a combined design of mindfulness and EI appears to carry greater benefits for adolescents (Mestre et al., 2019; Piqueras et al., 2020), and despite research being mainly centered on both independently, EI seems to clarify the relationship between mindfulness and both dependent variables through a harmonization between mindfulness mechanisms and the nature of EI cognitive processes. The mediation results also add evidence to recent empirical findings highlighting that mindfulness training may be favored from those with higher emotion regulation competencies (Magalhães et al., 2022), strengthening the importance of jointly explore the concepts as interactive and preventive factors of negative mental health in educational context. Regarding the relationship between mindfulness and well-being, no variations across gender have been noted, contradicting h2 in our study case, and previous findings suggesting this connection to be greater for females (e.g., Bluth et al., 2017; Carsley et al., 2018; Moreira et al., 2018). However, the literature fails consistency on this matter as other studies have not reported variations (e.g., Johnson et al., 2017; Magalhães et al., 2022). These results might indicate that emotion-regulatory patterns function similarly for male and female adolescents, which encourages future interventions to be designed with no distinction of individual characteristics. Still, these results must be interpreted with caution as they reinforce the need of replication and additional evidence of gender moderation effects.

The contributions of our study, however, should be observed in light of several methodological limitations. We highlight that causality conclusions could not be drawn due to the use of cross-sectional data thus future longitudinal studies are strongly recommended. Also worth of attention when looking through our results is the possibility of other predictor variables which have not been under analysis in our study impact our findings. Future research should consider a broader range of external elements, for example socioeconomic factors, school course and parents’ education level. In addition, the small sample size impacted the generalization of results. Moreover, the self-report nature of the instruments based on dispositional models may have implications, in addition to social desirability. The TEIQue-ASF has been theoretically linked to personality traits and parallelly discussed as a form of intelligence (Petrides & Furnham, 2001), and our study was centered on global trait EI. Future studies should consider a blended approach of ability and self-report measures. Furthermore, our study was unable to report an acceptable model fit for this measure, which compels the need of additional validation specifically addressing adolescent samples. Mavroveli and Siu (2012) previously reported cross-cultural fluctuation in psychometric indices of TEIQue-ASF, possibly related to the fact that the adolescent form falls in between the child version and the adult version of the measure, which has been considered to cause discrepancy in the perception of emotional information (p. 575). This observation was also highlighted by Feher and colleagues (2019). The study of Walter and colleagues (2021), suggested that both well-being and trait EI are comprised of different elements in different cultures which influence the constructs’ understanding. Similarly, validation studies of CAMM have identified structural discrepancies between cultures and populations (Limpo et al., 2022), despite its wide use in research. In addition, it should be alerted the possibility of students’ level of motivation to fulfill the questionnaire to be limited, and disengagement to increase over time with its length, so it could be relevant to follow-up with qualitative studies for more conclusive insights. Although we note that we were not able to observe strong correlations and we failed to report relationships between variables which have been pre-acknowledged in the literature, it is pertinent to take into account that our study is grounded on an exploratory nature rather than confirmatory. Our intention is to help sharpen future research and encourage the scientific community to deepener on such models and validate or contradict our findings. Despite the limitations, this study contributes towards the clarification of the relationship between mindfulness and EI, as trait-like constructs, and highlights the need of further exploring gender differences. Our findings are in line with the need of cultivating the debate on how can education move beyond technical learning, for instance throughout practical interventions, and confirms that a jointly approach to mindfulness and EI seems more advantageous than a separate strategy. The results suggest that fostering socioemotional competencies may have positive implications in educational settings, reinforcing the importance of adapting school curricula to include those elements, which can be particularly relevant in secondary school for encouraging emotional health in a period of higher propensity to lower psychological well-being.

## Data Availability

The data that support the findings of this study are available from the corresponding author upon reasonable request.
